# Commentary: Augmentation of Fear Extinction by Transcranial Direct Current Stimulation (tDCS)

**DOI:** 10.3389/fnbeh.2018.00121

**Published:** 2018-06-28

**Authors:** Rany Abend, Mascha van 't Wout

**Affiliations:** ^1^Emotion and Development Branch, National Institute of Mental Health, Bethesda, MD, United States; ^2^Department of Psychiatry and Human Behavior, Alpert Brown Medical School, Brown University, Providence, RI, United States; ^3^Center for Neurorestoration and Neurotechnology, Providence VA Medical Center, Providence, RI, United States

**Keywords:** stimulation, tDCS, extinction, fear, PTSD, anxiety

In their recent study, Dittert et al. examined whether transcranial direct current stimulation (tDCS) can facilitate fear extinction learning, as a preliminary experimental test toward future application of this technology for the enhancement of exposure-based therapy for anxiety and stress-related disorders. Conducted on a sample of healthy adults, Dittert et al. contrasted the effects of active vs. sham stimulation and right-anodal vs. left-anodal electrode alignment in a 1-day fear acquisition and extinction paradigm, with stimulation starting immediately after acquisition and continuing throughout extinction. The results indicate reduced physiological responding to the fear-conditioned stimulus (CS+) in the first two extinction trials, as well as increased responding to the non-reinforced safety stimulus (CS-) during early and late extinction associated with active stimulation.

We commend Dittert et al. for contributing to the growing literature on the application of neurostimulation methodologies for the improvement of psychopathology treatment, a research field that is experiencing a sharp rise in publications in recent years. In addition to the importance of the reported findings, this study offers the opportunity to critically highlight several aspects of the experimental application of neurostimulation techniques, and tDCS in particular, for the modulation of fear extinction process specifically, and cognitive/emotional functions in general. We believe that integrating these aspects in future studies may benefit research in this rapidly-growing and evolving field.

The first point concerns the implementation of fear conditioning and extinction (FCE) paradigms as models for fear learning and exposure-based therapy. In line with an established translational framework on the use of FCE paradigms (Milad et al., 2014), Dittert et al. employ a paradigm previously shown to lead to fear acquisition and extinction effects. However, several characteristics of FCE paradigms are important to consider in order to draw firmer conclusions on the effects of neurostimulation on fear extinction learning as they translate to the clinic.

First, administering the conditioning and extinction phases on separate days increases ecological validity, by providing a more appropriate neurobiological model for real-world traumatic events and their exposure-based treatment which are almost always separated by weeks to years. Temporally separating conditioning and extinction allows for some consolidation of the acquired fear memory to occur prior to extinction. In contrast, paradigms in which extinction immediately follows conditioning do not allow for fear memory consolidation (McGaugh, 2000), calling into question what is being modulated by stimulation—fear acquisition consolidation or extinction learning. Such 1-day paradigms may in fact be viewed more along the lines of reversal paradigms in which acquired contingencies are changed within the same session, and may thus potentially engage neural circuits other than those mediating extinction learning (Atlas et al., 2016). Second, the inclusion of an extinction recall (or test) phase in a following lab visit is vital in order to assess stimulation-induced modulation of fear extinction on the expression of the acquired and extinguished fear memory. That is, the dependent measure of FCE paradigms should be the effect on subsequent return of fear, akin to the magnitude of fear aroused when encountering a fear-evoking stimulus following the completion of exposure therapy.

Another issue pertains to reporting of unintended effects of stimulation which may be detrimental in a clinical context. The application of stimulation in the clinic is clearly intended to affect neural processes in a manner that results in beneficial outcomes. However, while theory often guides the hypotheses and subsequent operationalization of stimulation parameters (e.g., chosen electrode alignment targeting a specific neural system), stimulation may also inadvertently lead to effects that may be clinically undesirable. An example of this is the apparent stimulation-induced generalization of fear to the never conditioned, safe CS- that was observed by Dittert et al., as well as in studies by the authors (Abend et al., 2016; van't Wout et al., 2017). This observation indicates that stimulation may indeed modulate the targeted neural processes, but in a manner that is clinically detrimental, warranting further research before clinical implementation. Thus, negative effects of stimulation on clinically-relevant indices should be emphasized in studies alongside the beneficial effects, preferably with potential explanations as to why these negative effects might arise to inform future studies.

A final issue involves target engagement by stimulation. The effects of tDCS are relatively diffuse and current flow within the brain is complex, which is further complicated by the specific electrode montage used as well as intrinsic brain activity. Thus, it is not clear if the targeted neural circuitry is indeed engaged—or modulated in the intended direction—by stimulation and whether other neural or cognitive systems are collaterally affected. While current flow modeling software can be used to estimate the effect of stimulation on target regions, the use of concurrent imaging to monitor the effects of stimulation may critically aid in target validation, or alignment optimization, especially when target areas may not be directly proximal to the active electrodes (Saiote et al., 2013; Bergmann et al., 2016). Clearly, the integration of brain imaging methods incurs considerable costs and may not be readily available for all study centers. Nevertheless, when possible, a tDCS-imaging pilot study may provide information on target validation, critically informing the subsequent complete study design. Of note, multisite collaborative efforts may aid in this regard, allowing for sharing of resources and costs and for greater generalization of study findings.

In conclusion, the study by Dittert et al. provides an example for the experimental application of tDCS in the context of potentially enhancing exposure-based treatment for anxiety and stress-related disorders. At the same time, it also allows for the opportunity to highlight several aspects of tDCS research in the clinical context, as well as in the broader basic science context, which we hope would benefit future research in this growing field.

## Author contributions

All authors listed have made a substantial, direct and intellectual contribution to the work, and approved it for publication.

### Conflict of interest statement

The authors declare that the research was conducted in the absence of any commercial or financial relationships that could be construed as a potential conflict of interest.
